# Menstrual Cycle, Psychological Responses, and Adherence to Physical Exercise: Viewpoint of a Possible Barrier

**DOI:** 10.3389/fpsyg.2021.525943

**Published:** 2021-02-18

**Authors:** Raul Cosme Ramos Prado, Rodrigo Silveira, Marcus W. Kilpatrick, Flávio Oliveira Pires, Ricardo Yukio Asano

**Affiliations:** ^1^Women's Science Studies and Research Academy, São Paulo, Brazil; ^2^Exercise Psychophysiology Research Group, School of Arts, Sciences and Humanities, University of São Paulo, São Paulo, Brazil; ^3^School of Physical Education and Sport, University of São Paulo, São Paulo, Brazil; ^4^College of Education, University of South Florida, Tampa, FL, United States

**Keywords:** luteal phase, women, adherence to physical exercise, viewpoint, barrier, affect

## Introduction

The World Health Organization (WHO) warns that physical inactivity increases the number of global public health problems, such as the risk of non-communicable diseases (e.g., hypertension, diabetes, and cancer), stroke, heart attack, and mortality (World Health Organization, 2010). Alarming data from WHO indicate that about a quarter of the world's population is insufficiently active with a higher incidence in women (34%) than men (28%) in 2008 (World Health Organization, 2014) and 2016, 32 and 23%, respectively (World Health Organization, 2016). In 2018, a paper of *The Lancet Global Health* showed that women are about 8% more physically inactive (Guthold et al., 2018). Recognizing that many physically inactive adults have simply not initiated physical exercise programs, many other individuals are currently inactive after dropping out of exercise in the weeks and months of physical activity program initiation.

Recently, it has been shown that most people dropped out from regular physical exercise in the first few months (Withall et al., 2011; Sperandei et al., 2016). Sperandei et al. (2016) indicated that the dropout rate will reach more than half (i.e., 63%) of new exercise practitioners, from which only 4% will adhere to exercise beyond 12 months. Such concerns related to dropout seem especially important among women (Bennie et al., 2019). Also notable is research suggesting that women are motivated by exercise that produces pleasant feelings (Brickman and Campbell, 1971; Kahneman et al., 1999; Anderson et al., 2014; van Uffelen et al., 2017). Such motivations are highly relevant given the alterations to mood state linked to the menstrual cycle (MC).

In the premenstrual period, the hormonal fluctuations trigger several neural mechanisms that bring about physical (e.g., pain, swelling) and psychological (e.g., negative affect and mood) symptoms (Hellstrom and Anderberg, 2003; Ossewaarde et al., 2010; van Wingen et al., 2011). This psychological measure (e.g., positive affect) contributes to permanence in an activity (e.g., physical exercise). However, although the literature shows a strong relationship of dose–response between pleasant feelings and adherence, data generated about MC impact to the exercise adherence is still limited and, generally, does not consider the differences between the sexes and MC phases. Therefore, the present viewpoint highlights possible psychological barriers that the MC can generate in the adherence of women to physical exercise.

## Menstrual Cycle and Psychological Responses

Among the earliest studies on the MC were conducted by Robert Frank in the mid-1930s (Frank, 1931); from then on, studies have been showing that MC is regulated by the neuroendocrine system, specifically the hypothalamic–pituitary–ovarian (HPO) axis (Frank, 1931; Davis, 1979; Kubota et al., 2016). The MC lasts ~28 days and is divided into the follicular phase (FP; 1st−14th day) and the luteal phase (LP; 15th−28th day). While the FP comprises menstruation, follicle formation, high gonadotropin-releasing hormone, and estrogen (E_2_) concentration, the LP is characterized by the corpus luteum formation, the premenstrual period, and high concentrations of progesterone (P_4_). Also noteworthy is that the transition from FP to LP corresponds to peak levels of follicle-stimulating hormone and luteinizing hormone (Wilcox et al., 2000; Oyelowo, 2007; Selgrade et al., 2009).

A systematic review and meta-analyses indicated that studies published between 1980 and 2013 determined that psychological alterations are very common in women (Steel et al., 2014). The MC is frequently related to a negative change in the psychological status (Costello et al., 2014; Steel et al., 2014; Sundström Poromaa and Gingnell, 2014; Sundstrom-Poromaa, 2018), mainly during the LP (Ossewaarde et al., 2010; van Wingen et al., 2011). Ossewaarde et al. (2010) demonstrate that women had a greater sensibility to extrinsic stress and negative affect during the LP. The psychological changes in the LP are commonly associated with the action of gonadal hormones, neuroactive steroids, and/or sensibility to a stressor in the neurocircuitry that regulates emotions. However, there are different approaches to understanding these changes.

The first approach indicates that E_2_ and P_4_ easily cross the blood–brain barrier (Bixo et al., 1995, 1997) to connect to membrane-bound receptors (Murphy and Segal, 1996; Tang et al., 2004; Brinton et al., 2008), increasing reactivity in the prefrontal cortex (PFC) and amygdala in both animals (Murphy and Segal, 1996; Womble et al., 2002; Tang et al., 2004; Hao et al., 2006) and humans (McEwen and Woolley, 1994; Foy et al., 1999). The LP (i.e., lower E_2_ and higher P_4_) compared to FP (i.e., higher E_2_ and lower P_4_) showed a decrease in the PFC function combined with high amygdala reactivity (van Wingen et al., 2008; Pletzer et al., 2019). Hence, due to the important role of P_4_, the reduced PFC ability to inhibit negative amygdala responses during LP opens a vulnerability window increasing negative psychological responses (Craig, 2002; Phelps and LeDoux, 2005; Andreano and Cahill, 2010).

The second approach is related to the worst feelings of emotional memory in the LP. For example, Sabin and Slade (1999) showed that their participants reported negative emotions when they remembered LP. This has been associated with the negative impact of both P_4_ and cortisol from the HPA–adrenal cortex pathway (Ferin, 1996; Sabin and Slade, 1999; Herman et al., 2003; Sundström Poromaa and Gingnell, 2014). Several pieces of evidence agree that while the E_2_ acts passively on amygdala (Bayer et al., 2014), the P_4_ (Frye, 2007) and cortisol (Herman and Cullinan, 1997; Buchanan et al., 1999; Roozendaal, 2000; McGaugh and Roozendaal, 2002; Young, 2004) increase excitability and enhance the amygdala communication with medial PFC (mPFC) (van Wingen et al., 2011), which orchestrate a mechanism of anticipatory responses to psychological memories pleasant/unpleasant according to previous experiences (Roozendaal, 2000; Knutson et al., 2001; McGaugh and Roozendaal, 2002; Porro et al., 2002; Ochsner et al., 2005; Ertman et al., 2011). Thereby, this approach induces to suppose that the chronic rescue of unpleasant emotions in the LP can trigger a cyclical rhythm of negative anticipatory responses in women's life.

The third approach expresses negative emotions by the action of neuroactive steroids, such as allopregnanolone, on the GABA-ergic system inhibition (Andreen et al., 2006, 2009; Backstrom et al., 2011). The allopregnanolone level is lower during FP (~1.50 nmol/L) than in the LP (~7.00 nmol/L) (Bičíkov et al., 1995; Genazzani et al., 1998), similarly to P_4_ behavior (see previous approach), which is key in the neurosteroidogenesis of allopregnanolone (see Genazzani et al., 1998 for details).

Considering that the GABA-ergic system facilitates the main inhibition mechanism in the brain structures that process emotions (van Wingen et al., 2011), the allopregnanolone acts to enhance the hyperpolarization and, consequently, the inhibition effect of GABA receptors (Melcangi et al., 2011). However, the paradoxical effect of dose–response of allopregnanolone can be observed. While the low allopregnanolone level (e.g., FP) generates anxiolytic feelings, similarly to barbiturates and benzodiazepines to control calmness, anxiety, and other negative emotions, the high level (e.g., LP) impairs these characteristics and increases negative feelings (Kask et al., 2008; Backstrom et al., 2011; Melcangi et al., 2011).

The last approach indicates higher stress sensitivity during LP. Previous studies showed that stress induction potentiates negative mood change (Sabin and Slade, 1999) and negative affect (Ossewaarde et al., 2010) during LP. Similarly to the daily external stress (e.g., physical exercise) (Hill et al., 2008), the mechanism of induced stress (laboratory models) describes the GnRH inhibition from hypophysis by the hypothalamus and increases the corticotrophin-releasing hormone, adrenocorticotrophic hormone, and cortisol by the adrenal cortex, respectively (Ferin, 1999; Dobson and Smith, 2000). This mechanism triggers regions of the limbic system (e.g., arcuate nucleus and amygdala), negatively changing the mood and affective responses (Herman and Cullinan, 1997; Buchanan et al., 1999; Young, 2004) mainly during the LP (Sabin and Slade, 1999; Ossewaarde et al., 2010). However, this approach is complex and can impact directly the mechanisms cited above (see Sabin and Slade, 1999; Ossewaarde et al., 2010 and previous approaches).

A literature review report, about half of the female population experience mild physical and psychological premenstrual symptoms, about one fourth experience moderate symptoms (premenstrual syndrome; PMS), and about 8% experienced severe symptoms (premenstrual dysphoric disorder; PMDD) (Frackiewicz and Shiovitz, 2001). More recent data suggest higher incidence rates, with 35 and 10% of women officially diagnosable with PMS and PMDD, respectively (Alek et al., 2004).

These data indicate that sensitivity to act on psychological mechanisms differs among women, which can generate predominant or neutral effects of psychological change. It is also possible to consider that women diagnosed with PMS and PMDD will not necessarily have negative psychological alterations between MC phases. For example, a recent study (Prado et al., 2021) indicate that healthy women who are physically active and not utilizing exogenous hormones had lower motivation and affective responses before and during exercise in the LP compared to the FP. To conclude, we need to consider (i) that all psychological mechanisms are modulated by the sensibility level of neural receptors (Rubinow et al., 1998) and by the concentration of hormones and neuroactive steroids; (ii) the capacity of each woman to cushion the negative impacts (e.g., by the resilient personability) generated in the LP (Davydov et al., 2004).

Fundamentally, the above review highlights that women are naturally exposed to several physiological and psychological alterations, mainly during the premenstrual period. Once a vulnerability window of negative emotion is identified within the LP, it is possible to extrapolate that this period can negatively impact interest in activities of daily living and perhaps exercise. This can be an important step toward improving the understanding of adherent behavior in women. A related factor is that exercise occurring during the LP is more likely to be perceived as fatiguing and to result in reduced exercise capacity when compared to exercise during the FP (Freemas et al., 2020).

Established and emerging research make it clear that exercise adherence is related to psychological responses to exercise (Brickman and Campbell, 1971; Kahneman et al., 1999; Williams, 2008; Anderson et al., 2014). Less clear based on available research is how the MC might impact acute psychological responses and exercise adherence. This deficit in the research literature is especially important given the known psychological changes associated with the LP. Therefore, several hypotheses can be generated against this backdrop of limitations.

### Is the Menstrual Cycle a Barrier to Physical Exercise Adherence?

Bennie et al. (2019) observed that women were less likely to meet the adherence guidelines than men (Bennie et al., 2019). Overall, high social interaction and enjoyment can increase exercise adherence (Withall et al., 2011). Some studies have indicated that there are several motivators for exercise adherence that may vary between sexes (van Uffelen et al., 2017; Cañamero et al., 2019). For women, being able to meet friends, spending time with others, and feeling good were among the main factors, showing that the practice of exercise by women is motivated by pleasant feelings (van Uffelen et al., 2017).

On the other hand, several studies showed a decrease in negative feelings (i.e., affect, mood, anxiety) throughout the MC after an exercise training program (McDonald and Hodgdon, 1991; Petruzzello et al., 1991; Aganoff and Boyle, 1994). Aganoff and Boyle (1994) compared the effects of exercise on negative feelings throughout the MC between physically active and inactive women. Although they confirmed that the physically active group had more positive feelings compared to the physically inactive group, they also reported a high rate (i.e., about 20%) of sample loss during the study, which occurred exclusively in the physically active group. Although the authors provided a limited discussion about the dropout rate, we contend that these women may have dropped out of exercise during and/or because of the LP.

Researchers seem to agree that the premenstrual period interferes with physical activities (Hylan et al., 1999; Schneider et al., 1999; Ferrero et al., 2006) and perhaps in performance (Julian et al., 2017). Likewise, others showed a decrease of psychological tolerance during exercise (i.e., a high rating of perceived exertion; RPE) in the LP, especially caused by core temperature, P_4_, and cortisol (Pivarnik et al., 1992; Travlos and Marisi, 1996; Reilly, 2000; Janse de Jonge et al., 2012) that trigger some of the psychological mechanisms cited above (see section Menstrual Cycle and Psychological Responses). Also notable is that increases in stress hormones due to large doses of high-intensity exercise (Hill et al., 2008) can worsen feelings during the premenstrual period. As such, we suggest that negative emotions during LP can impair decision making, thus decreasing the tolerance for and interest in a lasting activity routine, such as adherence to physical exercise.

Recent research (Prado et al., 2021) of our group indicate lower motivation and affective responses before and during exercise in the LP compared to FP, in addition to higher RPE during exercise. Previous studies demonstrated a similar relationship between the increase in RPE and decrease in affective responses across continuous exercise (Ekkekakis et al., 2008) and the decrease of affective responses based on an increase of ventilatory rate (Vasconcelos et al., 2019). It is notable that any change between these measures (i.e., exercise intensity, RPE, affective responses, motivation) can impact the others.

Affective responses have been studied in sports science using the *Feeling Scale* in several contexts, including strength training (Elsangedy et al., 2018), endurance and high-intensity interval training (Oliveira et al., 2013; Kilpatrick et al., 2015), and different age groups (Benjamin et al., 2012; Barnett, 2013; Kilpatrick et al., 2015; Elsangedy et al., 2018). These studies provide support for the idea that brain structures responsible for affective responses interpret the afferent feedback from group III and IV fiber during exercise (Ekkekakis, 2003; Ekkekakis and Acevedo, 2006) and that this basic construct of pleasure has an important role to promote adherence to exercise.

Considering theories about human behavior describing how exposure to tasks that generate positive feelings can motivate adherence to exercise (Brickman and Campbell, 1971; Kahneman et al., 1999; Anderson et al., 2014), several studies showed a relationship between affective responses and adherence. A systematic review (Rhodes and Kates, 2015) demonstrated that affective responses based on moderate exercise were effective in increasing future intention to exercise. One study in this review observed that participants performing weekly physical exercise guided by their affective response showed that weekly total exercise time increased when participants self-selected the exercise intensity based on positive affective responses (Williams, 2008). Similarly, a pilot study investigating women's affective responses (Stevens et al., 2015) indicated that positive affect increased the intentions for future exercise.

Although some studies adopt several strategies to increase affective responses to exercise, such as music, video (Jones, 2015), and reduction of the exercise intensity throughout the session (Zenko et al., 2016), several psychological responses, including affect, are drastically reduced in the LP of the MC (Prado et al., 2021). Therefore, the consideration that the MC is a natural barrier for women's adherence to physical exercise is quite reasonable. Additionally, it is possible that these psychological impacts would be especially salient in women diagnosed with PMS or PMDD, who exhibit high emotional sensitivity, and those that receive hormone replacement.

**Figure 1:**
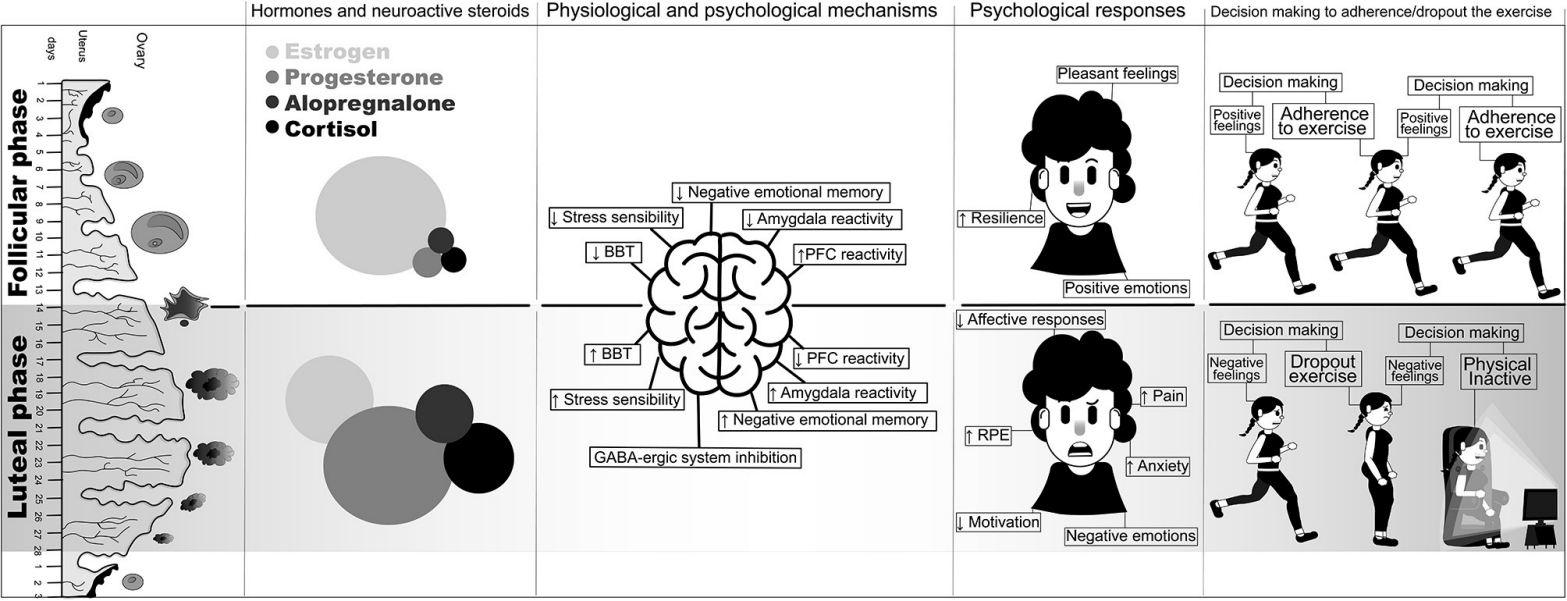
Schematic illustration of the possible barrier to adherence in the physical exercise by women. The uterus and ovary system; hormone and neuroactive steroids proportion; psychological responses and decision making to adherence/dropout exercise in the follicular (above) and luteal phase (bellow). BBT, basal body temperature; PFC, prefrontal cortex; RPE, rating of perceived exertion.

In conclusion, despite the fact that the extrapolation described here was based on independent data, we believe that the extrapolations made here provide a rational and important way to consider the prevalence of exercise dropout in women (Figure 1). Therefore, we suggest that future studies should consider the statements established in this viewpoint (e.g., LP–negative affect) and develop strategies, with experimental data, to confirm the cause–effect hypothesis between LP and dropout of the exercise.

### Future Perspectives for Research on Women's Adherence to Physical Exercise

We hope the ideas presented within this paper about the MC and adherence to physical exercise will be considered and facilitate optimization of future studies. Appropriate future research that seems appropriate include (i) follow-up studies that will identify the dropout prevalence between MC phases; (ii) studies that will demonstrate the effects of MC phases on predictors of adherence (e.g., affect); (iii) studies that evaluate the impact of hormone replacement therapy, diagnosis of PMS or PMDD, and high emotional sensitivity; and (iv) studies that will evaluate the physiological and psychological mechanisms associated that underpin the observations described within this paper.

Considering the women's particularities highlighted in this viewpoint, we also suggest that the extrapolation of data from previous studies should be cautious, mainly because these studies did not control the MC phases and/or inserted both sex simultaneously in their statistical analyses (Hall et al., 2007; Ekkekakis et al., 2008; Bellezza et al., 2009; Dasilva et al., 2011; Gerber et al., 2018, 2019; Saanijoki et al., 2018; Astorino and Sheard, 2019; Jones and Ekkekakis, 2019; Rhodewalt et al., 2019). Notably, it is important to recognize that the present paper exclusively discussed the impact of a biological factor on adherence to exercise and that, in addition to the MC, there are other factors (e.g., social environment, pregnancy, daily tasks, etc.) that can influence the adherent behavior.

Lastly, we assert that the questions raised here are important considerations within the context of global health promotion because a better understanding of how to optimize exercise experiences can reduce the burden of physical inactivity.

## Conclusion

In summary, the present letter calls attention to the impact of the MC on negative psychological changes, mainly in the luteal phase. Therefore, we suggest that the prevalence of physical inactivity and women's exercise dropout are due in part to pleasure being impaired during the premenstrual period. Ongoing investigations of these issues can facilitate the development of best practice strategies that will promote exercise behavior and improve health outcomes among women.

## Author Contributions

RP conceived the study. English reviewed by MK. All authors contributed to the writing of the manuscript and approved the final version, have made a substantial, direct and intellectual contribution to the work, and approved it for publication.

## Conflict of Interest

The authors declare that the research was conducted in the absence of any commercial or financial relationships that could be construed as a potential conflict of interest.

## References

[B1] Aganoff J. A. Boyle G. J. (1994). Aerobic exercise, mood states and menstrual cycle symptoms. J. Psychosom. Res. 38, 183–192. 10.1016/0022-3999(94)90114-7 8027958

[B2] Alek I. Lazarov A. Cordoba M. Zeitune M. Abraham D. Seidman D. (2004). Premenstrual syndrome and associated skin diseases related to hypersensitivity to female sex hormones. J. Reprod. Med. 49, 195–199. 15098889

[B3] Anderson N. D. Damianakis T. Kroger E. Wagner L. M. Dawson D. R. Binns M. A.. (2014). The benefits associated with volunteering among seniors: a critical review and recommendations for future research. Psychol. Bull. 140, 1505–1533. 10.1037/a0037610 25150681

[B4] Andreano J. M. Cahill L. (2010). Menstrual cycle modulation of medial temporal activity evoked by negative emotion. Neuroimage 53, 1286–1293. 10.1016/j.neuroimage.2010.07.011 20637290 PMC3376005

[B5] Andreen L. Nyberg S. Turkmen S. van Wingen G. Fernandez G. Backstrom T. (2009). Sex steroid induced negative mood may be explained by the paradoxical effect mediated by GABAA modulators. Psychoneuroendocrinology 34, 1121–1132. 10.1016/j.psyneuen.2009.02.003 19272715

[B6] Andreen L. Sundstrom-Poromaa I. Bixo M. Nyberg S. Backstrom T. (2006). Allopregnanolone concentration and mood–a bimodal association in postmenopausal women treated with oral progesterone. Psychopharmacology 187, 209–221. 10.1007/s00213-006-0417-0 16724185

[B7] Astorino T. A. Sheard A. C. (2019). Does sex mediate the affective response to high intensity interval exercise? Physiol. Behav. 204, 27–32. 10.1016/j.physbeh.2019.02.005 30738970

[B8] Backstrom T. Haage D. Lofgren M. Johansson I. M. Stromberg J. Nyberg S.. (2011). Paradoxical effects of GABA-A modulators may explain sex steroid induced negative mood symptoms in some persons. Neuroscience 191, 46–54. 10.1016/j.neuroscience.2011.03.061 21600269

[B9] Barnett F. (2013). The effect of exercise on affective and self-efficacy responses in older and younger women. J. Phys. Act. Health. 10, 97–105. 10.1123/jpah.10.1.97 22396245

[B10] Bayer J. Schultz H. Gamer M. Sommer T. (2014). Menstrual-cycle dependent fluctuations in ovarian hormones affect emotional memory. Neurobiol. Learn. Mem. 110, 55–63. 10.1016/j.nlm.2014.01.017 24492058

[B11] Bellezza P. A. Hall E. E. Miller P. C. Bixby W. R. (2009). The influence of exercise order on blood lactate, perceptual, and affective responses. J. Strength Cond. Res. 23, 203–208. 10.1519/JSC.0b013e3181889156 19130645

[B12] Benjamin C. C. Rowlands A. Parfitt G. (2012). Patterning of affective responses during a graded exercise test in children and adolescents. Pediatr. Exerc. Sci. 24, 275–288. 10.1123/pes.24.2.275 22728418

[B13] Bennie J. A. De Cocker K. Teychenne M. J. Brown W. J. Biddle S. J. H. (2019). The epidemiology of aerobic physical activity and muscle-strengthening activity guideline adherence among 383,928 U.S. adults. Int. J. Behav. Nutr. Phys. Act. 16:34. 10.1186/s12966-019-0797-2 30999896 PMC6472085

[B14] Bičíkov,á M. Lapčík O. Hampl R. Stárka L. Knuppen R. Haupt O.. (1995). A novel radioimmunoassay of allopregnanolone. Steroids 60, 210–213. 10.1016/0039-128X(94)00039-F 7618187

[B15] Bixo M. Andersson A. Winblad B. Purdy R. H. Backstrom T. (1997). Progesterone, 5alpha-pregnane-3,20-dione and 3alpha-hydroxy-5alpha-pregnane-20-one in specific regions of the human female brain in different endocrine states. Brain Res. 764, 173–178. 10.1016/S0006-8993(97)00455-1 9295207

[B16] Bixo M. Backstrom T. Winblad B. Andersson A. (1995). Estradiol and testosterone in specific regions of the human female brain in different endocrine states. J. Steroid Biochem. Mol. Biol. 55, 297–303. 10.1016/0960-0760(95)00179-4 8541226

[B17] Brickman P. Campbell D. T. (1971). Hedonic Relativism and Planning the Good Society. New York, NY: Academic Press, 287–301.

[B18] Brinton R. D. Thompson R. F. Foy M. R. Baudry M. Wang J. Finch C. E.. (2008). Progesterone receptors: form and function in brain. Front. Neuroendocrinol. 29, 313–339. 10.1016/j.yfrne.2008.02.001 18374402 PMC2398769

[B19] Buchanan T. W. al'Absi M. Lovallo W. R. (1999). Cortisol fluctuates with increases and decreases in negative affect. Psychoneuroendocrinology 24, 227–241. 10.1016/S0306-4530(98)00078-X 10101730

[B20] Cañamero S. R. García-Unanue J. Felipe J. L. Sánchez-Sánchez J. Gallardo L. (2019). Why do clients enrol and continue at sports centres?. Sport Bus. Manag. Int J. 9, 273–283. 10.1108/SBM-10-2018-0077

[B21] Costello J. T. Bieuzen F. Bleakley C. M. (2014). Where are all the female participants in sports and exercise medicine research? Eur. J. Sport Sci. 14, 847–851. 10.1080/17461391.2014.911354 24766579

[B22] Craig A. D. (2002). How do you feel? Interoception: the sense of the physiological condition of the body. Nat. Rev. Neurosci. 3, 655–666. 10.1038/nrn894 12154366

[B23] Dasilva S. G. Guidetti L. Buzzachera C. F. Elsangedy H. M. Krinski K. De Campos W.. (2011). Psychophysiological responses to self-paced treadmill and overground exercise. Med. Sci. Sports Exerc. 43, 1114–1124. 10.1249/MSS.0b013e318205874c 21088625

[B24] Davis P. J. (1979). Ageing and endocrine function. Clin. Endocrinol. Metab. 8, 603–619. 10.1016/S0300-595X(79)80033-X 389493

[B25] Davydov D. M. Shapiro D. Goldstein I. B. (2004). Moods in everyday situations: effects of menstrual cycle, work, and personality. J. Psychosom. Res. 56, 27–33. 10.1016/S0022-3999(03)00602-0 14987961

[B26] Dobson H. Smith R. F. (2000). What is stress, and how does it affect reproduction? Anim. Reprod. Sci. 60–61, 743–52. 10.1016/S0378-4320(00)00080-4 10844239

[B27] Ekkekakis P. (2003). Pleasure and displeasure from the body: perspectives from exercise. Cogn. Emot. 17, 213–239. 10.1080/02699930302292 29715726

[B28] Ekkekakis P. Acevedo E. (2006). Affective responses to acute exercise: toward a psychobiological dose-response model, in Psychobiology of Physical Activity (Human Kine), 91–109. Available online at: https://www.who.int/publications/i/item/9789241599979 (accessed January 2020).

[B29] Ekkekakis P. Hall E. E. Petruzzello S. J. (2008). The relationship between exercise intensity and affective responses demystified: to crack the 40-year-old nut, replace the 40-year-old nutcracker! Ann. Behav. Med. 35, 136–149. 10.1007/s12160-008-9025-z 18369689

[B30] Elsangedy H. M. Machado D. G. D. A. S. Krinski K. Duarte D. O. Nascimento P. H. De Amorim Oliveira G. T.. (2018). Let the pleasure guide your resistance training intensity. Med. Sci. Sports Exerc. 50, 1472–1479. 10.1249/MSS.0000000000001573 29432325

[B31] Ertman N. Andreano J. M. Cahill L. (2011). Progesterone at encoding predicts subsequent emotional memory. Learn. Mem. 18, 759–763. 10.1101/lm.023267.111 22101178 PMC3222892

[B32] Ferin M. (1999). Stress and the reproductive cycle. J. Clin. Endocrinol. Metab. 84, 1768–1774. 10.1210/jcem.84.6.5367 10372662

[B33] Ferin M. J. (1996). The menstrual cycle: an integrative view. Reprod. Endocrinol. Surg. Technol. 1, 103–121.

[B34] Ferrero S. Abbamonte L. H. Giordano M. Alessandri F. Anserini P. Remorgida V.. (2006). What is the desired menstrual frequency of women without menstruation-related symptoms? Contraception 73, 537–541. 10.1016/j.contraception.2006.01.004 16627042

[B35] Foy M. R. Xu J. Xie X. Brinton R. D. Thompson R. F. Berger T. W. (1999). 17beta-estradiol enhances NMDA receptor-mediated EPSPs and long-term potentiation. J. Neurophysiol. 81, 925–929. 10.1152/jn.1999.81.2.925 10036289

[B36] Frackiewicz E. J. Shiovitz T. M. (2001). Evaluation and management of premenstrual syndrome and premenstrual dysphoric disorder. J. Am. Pharm. Assoc. 41, 437–447. 10.1016/s1086-5802(16)31257-8 11372908

[B37] Frank R. (1931). The hormonal causes of premenstrual tension. Arch. Neurol. Psychiatry. 26, 1053–1057. 10.1001/archneurpsyc.1931.02230110151009

[B38] Freemas J. A. Baranauskas M. N. Constantini K. Constantini N. Greenshields J. T. Mickleborough T. D.. (2020). Exercise performance is impaired during the mid-luteal phase of the menstrual cycle. Med. Sci. Sports Exerc. 53, 442–452. 10.1249/MSS.0000000000002464 32694375

[B39] Frye C. A. (2007). Progestins influence motivation, reward, conditioning, stress, and/or response to drugs of abuse. Pharmacol. Biochem. Behav. 86, 209–219. 10.1016/j.pbb.2006.07.033 16979750 PMC3613144

[B40] Genazzani A. R. Petraglia F. Bernardi F. Casarosa E. Salvestroni C. Tonetti A.. (1998). Circulating levels of allopregnanolone in humans: gender, age, and endocrine influences. J. Clin. Endocrinol. Metab. 83, 2099–2103. 10.1210/jcem.83.6.4905 9626145

[B41] Gerber M. Minghetti A. Beck J. Zahner L. Donath L. (2018). Sprint interval training and continuous aerobic exercise training have similar effects on exercise motivation and affective responses to exercise in patients with major depressive disorders: a randomized controlled trial. Front. Psychiatry 9:694. 10.3389/fpsyt.2018.00694 30622487 PMC6308196

[B42] Gerber M. Minghetti A. Beck J. Zahner L. Donath L. (2019). Is improved fitness following a 12-week exercise program associated with decreased symptom severity, better wellbeing, and fewer sleep complaints in patients with major depressive disorders? A secondary analysis of a randomized controlled trial. J. Psychiatr. Res. 113, 58–64. 10.1016/j.jpsychires.2019.03.011 30903972

[B43] Guthold R. Stevens G. A. Riley L. M. Bull F. C. (2018). Worldwide trends in insufficient physical activity from 2001 to 2016: a pooled analysis of 358 population-based surveys with 1·9 million participants. Lancet Glob. Health 6, e1077–e1086. 10.1016/S2214-109X(18)30357-7 30193830

[B44] Hall E. E. Ekkekakis P. Petruzzello S. J. (2007). Regional brain activity and strenuous exercise: predicting affective responses using EEG asymmetry. Biol. Psychol. 75, 194–200. 10.1016/j.biopsycho.2007.03.002 17449167

[B45] Hao J. Rapp P. R. Leffler A. E. Leffler S. R. Janssen W. G. M. Lou W.. (2006). Estrogen alters spine number and morphology in prefrontal cortex of aged female rhesus monkeys. J. Neurosci. 26, 2571–2578. 10.1523/JNEUROSCI.3440-05.2006 16510735 PMC6793646

[B46] Hellstrom B. Anderberg U. M. (2003). Pain perception across the menstrual cycle phases in women with chronic pain. Percept. Mot. Skills 96, 201–211. 10.2466/pms.2003.96.1.201 12705527

[B47] Herman J. P. Cullinan W. E. (1997). Neurocircuitry of stress: central control of the hypothalamo-pituitary-adrenocortical axis. Trends Neurosci. 20, 78–84. 10.1016/S0166-2236(96)10069-2 9023876

[B48] Herman J. P. Figueiredo H. Mueller N. K. Ulrich-Lai Y. Ostrander M. M. Choi D. C.. (2003). Central mechanisms of stress integration: hierarchical circuitry controlling hypothalamo-pituitary-adrenocortical responsiveness. Front. Neuroendocrinol. 24, 151–180. 10.1016/j.yfrne.2003.07.001 14596810

[B49] Hill E. E. Zack E. Battaglini C. Viru M. Viru A. Hackney A. C. (2008). Exercise and circulating cortisol levels: the intensity threshold effect. J. Endocrinol. Invest. 31, 587–591. 10.1007/BF03345606 18787373

[B50] Hylan T. R. Sundell K. Judge R. (1999). The impact of premenstrual symptomatology on functioning and treatment-seeking behavior: experience from the United States, United Kingdom, and France. J. Womens Health Gend. Based Med. 8, 1043–1052. 10.1089/jwh.1.1999.8.1043 10565662

[B51] Janse de Jonge X. A. K. Thompson M. W. Chuter V. H. Silk L. N. Thom J. M. (2012). Exercise performance over the menstrual cycle in temperate and hot, humid conditions. Med. Sci. Sports Exerc. 44, 2190–2198. 10.1249/MSS.0b013e3182656f13 22776870

[B52] Jones L. (2015). Can high-intensity exercise be more pleasant? attentional dissociation using music and video. J. Sport Exerc. Psychol. 37, 436–448. 25356615 10.1123/jsep.2013-0251

[B53] Jones L. Ekkekakis P. (2019). Affect and prefrontal hemodynamics during exercise under immersive audiovisual stimulation: Improving the experience of exercise for overweight adults. J. Sport Health Sci. 8, 325–338. 10.1016/j.jshs.2019.03.003 31333885 PMC6620430

[B54] Julian R. Hecksteden A. Fullagar H. H. K. Meyer T. (2017). The effects of menstrual cycle phase on physical performance in female soccer players. PLoS ONE 12:e0173951. 10.1371/journal.pone.0173951 28288203 PMC5348024

[B55] Kahneman D. Diener E. Schwarz N. (1999). Well-Being: Foundations of Hedonic Psychology. Russell Sage Foundation. p. 608. Available online at: http://www.jstor.org/stable/10.7758/9781610443258 (accessed January 2020).

[B56] Kask K. Backstrom T. Nilsson L.-G. Sundstrom-Poromaa I. (2008). Allopregnanolone impairs episodic memory in healthy women. Psychopharmacology 199, 161–168. 10.1007/s00213-008-1150-7 18551282

[B57] Kilpatrick M. W. Greeley S. J. Collins L. H. (2015). The impact of continuous and interval cycle exercise on affect and enjoyment. Res. Q. Exerc. Sport 86, 244–251. 10.1080/02701367.2015.1015673 25811234

[B58] Knutson B. Fong G. W. Adams C. M. Varner J. L. Hommer D. (2001). Dissociation of reward anticipation and outcome with event-related fMRI. Neuroreport 12, 3683–3687. 10.1097/00001756-200112040-00016 11726774

[B59] Kubota K. Cui W. Dhakal P. Wolfe M. W. Rumi M. A. K. Vivian J. L.. (2016). Rethinking progesterone regulation of female reproductive cyclicity. Proc. Natl. Acad. Sci. U. S. A. 113, 4212–4217. 10.1073/pnas.1601825113 27035990 PMC4839436

[B60] McDonald D. G. Hodgdon J. A. (1991). Recent research in psychology, in The Psychological Effects of Aerobic Fitness Training: Research and Theory. Springer-Verlag Publishing. 10.1007/978-1-4612-3182-0

[B61] McEwen B. S. Woolley C. S. (1994). Estradiol and progesterone regulate neuronal structure and synaptic connectivity in adult as well as developing brain. Exp. Gerontol. 29, 431–436. 10.1016/0531-5565(94)90022-1 7925761

[B62] McGaugh J. L. Roozendaal B. (2002). Role of adrenal stress hormones in forming lasting memories in the brain. Curr. Opin. Neurobiol. 12, 205–210. 10.1016/S0959-4388(02)00306-9 12015238

[B63] Melcangi R. C. Panzica G. Garcia-Segura L. M. (2011). Neuroactive steroids: focus on human brain. Neuroscience 191, 1–5. 10.1016/j.neuroscience.2011.06.024 21704130

[B64] Murphy D. D. Segal M. (1996). Regulation of dendritic spine density in cultured rat hippocampal neurons by steroid hormones. J. Neurosci. 16, 4059–4068. 10.1523/JNEUROSCI.16-13-04059.1996 8753868 PMC6578996

[B65] Ochsner K. Knierim K. Ludlow D. Hanelin J. Ramachandran T. Glover G.. (2005). Reflecting upon feelings: an fMRI study of neural systems supporting the attribution of emotion to self and other. J. Cogn. Neurosci. 16, 1746–1772. 10.1162/0898929042947829 15701226

[B66] Oliveira B. R. R. Slama F. A. Deslandes A. C. Furtado E. S. Santos T. M. (2013). Continuous and high-intensity interval training: which promotes higher pleasure? PLoS ONE 8:e79965. 10.1371/journal.pone.0079965 24302993 PMC3841165

[B67] Ossewaarde L. Hermans E. J. van Wingen G. A. Kooijman S. C. Johansson I.-M. Backstrom T.. (2010). Neural mechanisms underlying changes in stress-sensitivity across the menstrual cycle. Psychoneuroendocrinology 35, 47–55. 10.1016/j.psyneuen.2009.08.011 19758762

[B68] Oyelowo T. (2007). Chapter 3 - Menstrual Cycle. In: Oyelowo TBT-MG to WH, editor. Saint Louis: Mosby, 11–15.

[B69] Petruzzello S. Landers D. Hatfield B. Kubitz K. Salazar W. (1991). A meta-analysis on the anxiety-reducing effects of acute and chronic exercise. Sports Med. 11, 143–182. 10.2165/00007256-199111030-00002 1828608

[B70] Phelps E. A. LeDoux J. E. (2005). Contributions of the amygdala to emotion processing: from animal models to human behavior. Neuron 48, 175–187. 10.1016/j.neuron.2005.09.025 16242399

[B71] Pivarnik J. M. Marichal C. J. Spillman T. Morrow J. R. J. (1992). Menstrual cycle phase affects temperature regulation during endurance exercise. J. Appl. Physiol. 72, 543–548. 10.1152/jappl.1992.72.2.543 1559930

[B72] Pletzer B. Harris T.-A. Scheuringer A. Hidalgo-Lopez E. (2019). The cycling brain: menstrual cycle related fluctuations in hippocampal and fronto-striatal activation and connectivity during cognitive tasks. Neuropsychopharmacology 44, 1867–1875. 10.1038/s41386-019-0435-3 31195407 PMC6785086

[B73] Porro C. A. Baraldi P. Pagnoni G. Serafini M. Facchin P. Maieron M.. (2002). Does anticipation of pain affect cortical nociceptive systems? J. Neurosci. 22, 3206–3214. 10.1523/JNEUROSCI.22-08-03206.2002 11943821 PMC6757517

[B74] Prado R. C. R. Silveira R. Kilpatrick M. W. Pires F. O. Asano R. Y. (2021). The effect of menstrual cycle and exercise intensity on psychological and physiological responses in healthy eumenorrheic women. Physiol. Behav. 232:113290. 10.1016/j.physbeh.2020.113290 33333131

[B75] Reilly T. (2000). The menstrual cycle and human performance: an overview. Biol. Rhythm. Res. 31, 29–40. 10.1076/0929-1016(200002)31:1;1-0;FT029

[B76] Rhodes R. E. Kates A. (2015). Can the affective response to exercise predict future motives and physical activity behavior? A systematic review of published evidence. Ann. Behav. Med. 49:715–731. 10.1007/s12160-015-9704-5 25921307

[B77] Rhodewalt R. Saur B. Largent K. Astorino T. A. Zenko Z. Schubert M. M. (2019). Effect of fed state on self-selected intensity and affective responses to exercise following public health recommendations. Int J Exerc Sci. 12, 602–613. 31156742 10.70252/RYIR6699 PMC6533088

[B78] Roozendaal B. (2000). Glucocorticoids and the regulation of memory consolidation. Psychoneuroendocrinology 25, 213–238. 10.1016/S0306-4530(99)00058-X 10737694

[B79] Rubinow D. R. Schmidt P. J. Roca C. A. (1998). Estrogen-serotonin interactions: implications for affective regulation. Biol. Psychiatry 44, 839–850. 10.1016/S0006-3223(98)00162-0 9807639

[B80] Saanijoki T. Nummenmaa L. Koivumaki M. Loyttyniemi E. Kalliokoski K. K. Hannukainen J. C. (2018). Affective adaptation to repeated SIT and MICT protocols in insulin-resistant subjects. Med. Sci. Sports Exerc. 50, 18–27. 10.1249/MSS.0000000000001415 28857909

[B81] Sabin R. Slade P. (1999). Reconceptualizing pre-menstrual emotional symptoms as phasic differential responsiveness to stressors. J. Reprod. Infant. Psychol. 17, 381–390. 10.1080/02646839908404603

[B82] Schneider M. B. Fisher M. Friedman S. B. Bijur P. E. Toffler A. P. (1999). Menstrual and premenstrual issues in female military cadets: a unique population with significant concerns. J. Pediatr. Adolesc. Gynecol. 12, 195–201. 10.1016/S1083-3188(99)00025-X 10584223

[B83] Selgrade J. F. Harris L. A. Pasteur R. D. (2009). A model for hormonal control of the menstrual cycle: structural consistency but sensitivity with regard to data. J. Theor. Biol. 260, 572–580. 10.1016/j.jtbi.2009.06.017 19560471

[B84] Sperandei S. Vieira M. C. Reis A. C. (2016). Adherence to physical activity in an unsupervised setting: explanatory variables for high attrition rates among fitness center members. J. Sci. Med. Sport 19, 916–920. 10.1016/j.jsams.2015.12.522 26874647

[B85] Steel Z. Marnane C. Iranpour C. Chey T. Jackson J. W. Patel V.. (2014). The global prevalence of common mental disorders: a systematic review and meta-analysis 1980-2013. Int. J. Epidemiol. 43, 476–493. 10.1093/ije/dyu038 24648481 PMC3997379

[B86] Stevens C. Smith J. Bryan A. (2015). A pilot study of women's affective responses to common and uncommon forms of aerobic exercise. Psychol. Health 31, 1–33. 10.1080/08870446.2015.1095917 26394246 PMC4684981

[B87] Sundström Poromaa I. Gingnell M. (2014). Menstrual cycle influence on cognitive function and emotion processing—from a reproductive perspective. Front. Neurosci. 8:380. 10.3389/fnins.2014.00380 25505380 PMC4241821

[B88] Sundstrom-Poromaa I. (2018). The menstrual cycle influences emotion but has limited effect on cognitive function. Vitam. Horm. 107, 349–376. 10.1016/bs.vh.2018.01.016 29544637

[B89] Tang Y. Janssen W. G. M. Hao J. Roberts J. A. McKay H. Lasley B.. (2004). Estrogen replacement increases spinophilin-immunoreactive spine number in the prefrontal cortex of female rhesus monkeys. Cereb. Cortex 14, 215–223. 10.1093/cercor/bhg121 14704219

[B90] Travlos A. K. Marisi D. Q. (1996). Perceived exertion during physical exercise among individuals high and low in fitness. Percept. Mot. Skills 82, 419–424. 10.2466/pms.1996.82.2.419 8724910

[B91] van Uffelen J. G. Z. Khan A. Burton N. W. (2017). Gender differences in physical activity motivators and context preferences: a population-based study in people in their sixties. BMC Public Health 17:624. 10.1186/s12889-017-4540-0 28676081 PMC5496591

[B92] van Wingen G. A. Ossewaarde L. Bäckström T. Hermans E. J. Fernández G. (2011). Gonadal hormone regulation of the emotion circuitry in humans. Neuroscience 191, 38–45. 10.1016/j.neuroscience.2011.04.042 21540080

[B93] van Wingen G. A. van Broekhoven F. Verkes R. J. Petersson K. M. Backstrom T. Buitelaar J. K.. (2008). Progesterone selectively increases amygdala reactivity in women. Mol. Psychiatry 13, 325–333. 10.1038/sj.mp.4002030 17579609

[B94] Vasconcelos G. Canestri R. Prado R. C. R. Brietzke C. Franco-Alvarenga P. Santos T. M.. (2019). A comprehensive integrative perspective of the anaerobic threshold engine. Physiol. Behav. 210:112435. 10.1016/j.physbeh.2019.01.019 30685364

[B95] Wilcox A. J. Dunson D. Baird D. D. (2000). The timing of the “fertile window” in the menstrual cycle: day specific estimates from a prospective study. BMJ 321, 1259–1262. 10.1136/bmj.321.7271.1259 11082086 PMC27529

[B96] Williams D. M. (2008). Exercise, affect, and adherence: an integrated model and a case for self-paced exercise. J. Sport Exerc. Psychol. 30, 471–496. 10.1123/jsep.30.5.471 18971508 PMC4222174

[B97] Withall J. Jago R. Fox K. R. (2011). Why some do but most don't. Barriers and enablers to engaging low-income groups in physical activity programmes: a mixed methods study. BMC Public Health 11:507. 10.1186/1471-2458-11-507 21711514 PMC3141466

[B98] Womble M. D. Andrew J. A. Crook J. J. (2002). 17β-Estradiol reduces excitatory postsynaptic potential (EPSP) amplitude in rat basolateral amygdala neurons. Neurosci. Lett. 331, 83–86. 10.1016/S0304-3940(02)00871-6 12361846

[B99] World Health Organization (2010). Global Recommendations on Physical Activity for Health. Geneva World Health Organization, 60. Available online at: http://medcontent.metapress.com/index/A65RM03P4874243N.pdf%5Cnhttp://scholar.google.com/scholar?hl=en&btnG=Search&q=intitle:Global+Recomendations+on+physical+activity+for+health#0 (accessed January 2020).

[B100] World Health Organization (2014). Physical Inactivity: A Global Public Health Problem. WHO. Available online at: https://www.who.int/dietphysicalactivity/factsheet_inactivity/en/#.XUh-Qbhbf7M.mendeley (accessed August 5, 2019).

[B101] World Health Organization (2016). Global Health Observatory: Prevalence of Insufficient Physical Activity. World Health Organization. Available online at: http://www.who.int/gho/ncd/risk_factors/physical_activity_text/en/index.html (accessed January 2020).

[B102] Young A. H. (2004). Cortisol in mood disorders. Stress 7, 205–208. 10.1080/10253890500069189 16019585

[B103] Zenko Z. Ekkekakis P. Ariely D. (2016). Can you have your vigorous exercise and enjoy it too? Ramping intensity down increases postexercise, remembered, and forecasted pleasure. J. Sport Exerc. Psychol. 38, 149–159. 10.1123/jsep.2015-0286 27390185

